# The influence of intrathecal injection of methotrexate and dexamethasone on neuropsychiatric systemic lupus erythematosus (NPSLE): a retrospective cohort study of 386 patients with NPSLE

**DOI:** 10.1186/s13075-023-03030-w

**Published:** 2023-03-28

**Authors:** Yuxue Nie, Boyuan Sun, Xin He, Minmin Zheng, Di Wu, Yunjiao Yang, Li Zhang, Wei Bai, Nan Jiang, Lin Qiao, Can Huang, Shuang Zhou, Jiaxin Zhou, Linyi Peng, Jingwen Niu, Mengtao Li, Yan Zhao, Xiaofeng Zeng, Li Wang, Wen Zhang

**Affiliations:** 1 Department of Rheumatology, National Clinical Research Center for Dermatologic and Immunologic Diseases, State Key Laboratory of Complex Severe and Rare Diseases, Key Laboratory of Rheumatology and Clinical Immunology, Ministry of Education, Peking Union Medical College Hospital, Chinese Academy of Medical Science and Peking Union Medical College, Beijing, China; 2 Department of Hematology and Rheumatology, Zhongshan Boai Hospital Affiliated to Southern Medical University, Zhongshan, China; 3 Department of Neurology, Peking Union Medical College Hospital, Chinese Academy of Medical Sciences, Beijing, China

**Keywords:** Lupus erythematosus, Systemic, Neurologic manifestations, Therapy

## Abstract

**Background:**

Neuropsychiatric involvement is one of the major concerns in systemic lupus erythematosus (SLE). The therapeutic effect of intrathecal treatment of methotrexate and dexamethasone has been investigated in some exploratory studies, but its influence on the long-term prognosis of neuropsychiatric SLE (NPSLE) remains unknown.

**Methods:**

This was a propensity score-matched retrospective study. Outcomes at discharge and time free from NPSLE relapse or death were evaluated by multivariate logistic regression, survival analysis, and Cox regression as appropriate.

**Results:**

Among 386 hospitalized patients with NPSLE, the median [IQR] age was 30.0 [23.0–40.0] years, and 342 patients (88.4%) were female. Of those, 194 patients received intrathecal treatment. Patients in the intrathecal treatment group had higher Systemic Lupus Erythematosus Disease Activity Index 2000 scores (median 17 vs. 14 points, IQR 12–22 vs. 10–19 points, *P* <0 .001) and were more likely to receive methylprednisolone pulse therapy (71.6% vs. 49.5%, *P* < 0.001) than those who did not receive intrathecal therapy. Intrathecal treatment was associated with a higher probability of survival and being free from NPSLE relapse than control treatment among the 386 unmatched patients (*P* =0.042 by log-rank test) and within 147 propensity score-matched pairs (*P* =0.032 by log-rank test). In the subgroup of NPSLE patients with increased levels of protein in cerebrospinal fluid, intrathecal treatment had a positive influence on their prognosis (*P* < 0.001).

**Conclusions:**

Intrathecal treatment of methotrexate and dexamethasone was associated with a more favorable prognosis of NPSLE and may serve as a valuable additional therapy for NPSLE patients, especially for those with elevated levels of protein in cerebrospinal fluid.

**Supplementary Information:**

The online version contains supplementary material available at 10.1186/s13075-023-03030-w.

## Background

Systemic lupus erythematosus (SLE) is a chronic autoimmune disease involving multiple organ systems[1], of which the nervous system is often affected. The prevalence of neuropsychiatric SLE (NPSLE) was reported to be 17.6% in retrospective studies and 44.5% in prospective studies in a meta-analysis including 5057 SLE patients[2]. NPSLE encompasses a heterogeneous group of conditions, with 19 distinct subtypes previously defined by the American College of Rheumatology (ACR) in 1999[3]. Correspondingly, the pathogenesis of NPSLE also varies and generally falls into either immune/inflammatory or thrombotic/ischemic categories[4]. According to the EULAR recommendations for the management of NPSLE in 2010[5] and the 2019 update of the EULAR recommendations for the management of SLE[6], glucocorticoids and immunosuppressive agents were indicated for NPSLE patients with symptoms associated with an inflammatory or immune-mediated process such as psychosis, acute confusional state (ACS), and myelitis, while antiplatelet or anticoagulation therapy was recommended in cases related to antiphospholipid antibodies. Other treatments included symptomatic and supportive treatment as needed. Thus, systemic therapy, including immunosuppressive therapy and antiplatelet/anticoagulation as indicated, is the mainstay of NPSLE treatment.

However, given the heterogeneity of NPSLE, such treatment may not be sufficient, especially for severe or refractory cases, partly because of the limited penetration of therapeutic agents through the blood‒brain barrier into the central nervous system (CNS). In this scenario, intrathecal treatment may become a useful supplementary treatment strategy. Intrathecal injection of methotrexate (MTX) plus dexamethasone (DEX) was first reported in 1994 in three SLE patients with severe CNS involvement, and encouraging outcomes were observed[7]. Another study in 1999 reviewed 24 SLE patients with severe CNS involvement who received intrathecal injection of MTX and DEX in addition to oral glucocorticoids and CYC and were refractory to conventional treatment with methylprednisolone pulse therapy and CYC or considered to be not suitable for methylprednisolone pulse therapy[8], and those who received intrathecal treatment exhibited an overall response rate of 91.7% (22/24). In another retrospective study in 2006, 240 patients with NPSLE were included, and 109 of them received intrathecal treatment with MTX and DEX[9]. In the logistic regression analysis, intrathecal treatment was associated with a better outcome of NPSLE (adjusted OR 0.41, 95% CI 0.11–0.87,* P* = 0.010), defined as improvement at discharge based on the modified Neuwelt classification[10]. Despite the relatively large number of patients in this study, there was no follow-up information, and thus, the long-term influence of intrathecal treatment on NPSLE was unknown. Another small-size study included 36 NPSLE patients treated with MTX and DEX intrathecally and 36 SLE patients without neuropsychiatric symptoms as the control group, where all patients had steroid pulse treatment[11]. After treatment, 77.8% (28/36) of the NPSLE cases were in clinical remission, 11.1% (4/36) of the cases were effectively treated, and 11.1% (4/36) of the cases were invalid, with a total effective rate of 88.9%. However, the simultaneous use of a large dose of glucocorticoids with intrathecal treatment made it difficult to determine the role of intrathecal therapy exactly. In summary, the previous studies of intrathecal treatment were exploratory to a large extent; the biases were not well controlled, and the evaluation of patient outcomes varied without long-term follow-up.

Thus, current evidence on intrathecal therapy in NPSLE is still limited, and it was not recommended as a common therapy in the 2010 EULAR recommendation for the management of NPSLE[5] or the 2019 update of the EULAR recommendations for the management of SLE[6]. Nevertheless, based on the nearly 30 years of experience with intrathecal treatment for NPSLE at Peking Union Medical College Hospital (PUMCH), a therapeutic role of intrathecal treatment, especially for those with severe and inflammatory or immune-mediated NPSLE or those refractory to standard immunosuppressive treatment, has been observed in real-world clinical practice.

Herein, in the present study, we aimed to summarize the experience of intrathecal treatment in a tertiary medical center in China, not only based on the outcome at discharge but also based on a relatively long follow-up period. Importantly, all the patients in the study, regardless of whether they received intrathecal treatment, underwent lumbar puncture, and CSF examinations were performed. In addition, a large proportion of the intrathecal treatment group and a part of the control group had CSF tests performed more than once, allowing us to compare the changes in CSF examination results between the two groups and to investigate the changes in CSF measurements after intrathecal injection of MTX and DEX in a longitudinal way. In addition, since the allocation of patients to receive intrathecal therapy or not was not randomized, the propensity score matching (PSM) method[12] was applied to construct a comparable cohort to minimize the discrepancy between those receiving intrathecal treatment and those who did not. We hope that this study will provide more efficacy and safety information about intrathecal treatment in NPSLE patients.

## Methods

### Patient selection

First, we screened 5604 patients with a final diagnosis of “systemic lupus erythematosus” hospitalized at PUMCH from January 2013 to June 2021. Then, we searched those cases with the keywords “lupus encephalopathy” and “neuropsychiatric lupus,” and 463 patients with possible NPSLE were identified. The medical records of these patients were reviewed systemically, and two experts in rheumatology and one expert in neurology discussed uncertain cases. Next, we excluded those patients who did not undergo lumbar puncture or had an uncertain diagnosis of NPSLE. Finally, 386 patients were included. All patients included fulfilled the 1997 American College of Rheumatology (ACR) classification criteria[13] or the 2012 Systemic Lupus International Collaborating Clinics (SLICC) Classification Criteria[14] for SLE. The diagnosis of NPSLE referred to the neurologic and psychiatric syndromes involving the nervous system categorized by the ACR subcommittee in 1999[3]. To ensure that the neuropsychiatric events were attributable to NPSLE instead of the other secondary causes, such as infection and metabolism disorders, we made the following efforts. First, all of the patients in this study were hospitalized; therefore, the patients were systemically and carefully screened for the other secondary causes by an experienced medical team during hospitalization. Specifically, tests for differentiating NPSLE from other secondary causes of neuropsychiatric events were performed, including blood pressure monitoring, blood laboratory tests (tests for blood levels of electrolytes, glucose, creatinine, and vitamin B12; anemia; blood gases; etc., as appropriate). In addition, neuroimaging, including magnetic resonance imaging (MRI), was conducted for all patients in the study if there were no contraindications. Otherwise, computed tomography (CT) scans of the brain were performed. Moreover, all 386 patients underwent lumbar punctures and had CSF examinations, including white blood cell count and classification; the measurement of protein, glucose, and chloride levels; and cytological analysis when needed. Tests for pathogens in CSF were performed in every patient, including smears with Gram, acid-fast, and India ink staining; cryptococcal antigen latex agglutination system tests; *Mycobacterium tuberculosis* DNA detection by polymerase chain reaction; and microorganism culture. Antibodies against viruses and parasites in CSF were tested when necessary. Moreover, if there was doubt about the NPSLE diagnosis in some patients after thorough history taking, physical examinations, and the tests mentioned above, a multidisciplinary discussion including experts in rheumatology, neurology, infectious diseases, and psychiatry was initiated. Regarding the subtypes of NPSLE, headache was defined by the International Headache Society (IHS) classification[15].

All neuropsychiatric events were new or worsened at baseline. Information on intrathecal treatment was collected retrospectively from medical records. Lumbar puncture and intrathecal treatment were performed after informed consent was acquired from the patients. This study was approved by the Ethics Committee of PUMCH (approval number: SK-1871).

### Data collection

The medical records of 386 patients were systematically reviewed, and all related clinical data, including demographic features, clinical manifestations, laboratory findings, Systemic Lupus Erythematosus Disease Activity Index 2000 (SLEDAI-2K) scores, and treatment, were collected. Follow-ups and outcomes were also recorded. All patients underwent lumbar puncture, and ICP and CSF examination results were collected as mentioned.

### Intrathecal therapy

MTX and DEX were used as standard reagents for intrathecal injection. However, there was approximately 1 year when the kind of MTX for intrathecal injection was unavailable at PUMCH, during which DEX was the only component used in intrathecal therapy. During intrathecal treatment, 10 mg of DEX with or without 10 mg of MTX was diluted with normal saline to a final volume of 10 ml before being injected intrathecally. CSF dilution was applied, and the process lasted no less than 5 min. One to six courses of intrathecal therapy were administered once per week to the intrathecal treatment group[8].

### Follow-up

After the last lumbar puncture, patients continued to be hospitalized for at least one week to observe potential adverse events. After discharge, patients were followed up every month for at least 3 months, and then the follow-up interval was prolonged if the disease condition was stable, as decided by the patients’ attending rheumatologists. In addition to the evaluation of the disease itself, any possible adverse events related to intrathecal therapy and lumbar puncture were also recorded.

### Outcome measures

Patient outcomes were defined from three aspects: outcome at discharge, change in CSF pressure and protein levels at the last lumbar puncture compared with baseline, and endpoint events (death from all causes or the relapse of NPSLE) during follow-up. The outcome at discharge was classified into three groups: complete recovery, partial improvement, and exacerbation. Complete recovery was defined as patients showing complete remission of NPSLE manifestations fulfilling grade D of the British Isles Lupus Assessment Group (BILAG)[16] and without residual neurological deficits. Partial improvement was defined as patients showing improved clinical outcomes but residual neurological deficits. Exacerbation included patients with exacerbated neuropsychiatric symptoms or those who died during hospitalization. For patients who underwent lumbar punctures more than once, ICP and CSF examination results were recorded every time. For the evaluation of long-term outcomes, we defined death from all causes or the relapse of NPSLE as an endpoint event during follow-up.

### Statistical analyses

PSM was performed to minimize the imbalance in the covariates between the patients who received intrathecal treatment and those who did not. Propensity scores (PSs) were calculated based on covariates that differed between the two groups and might lead to confusion on the influence of intrathecal treatment on the patients’ outcomes: sex, age, SLEDAI-2K scores, headache, psychosis, and methylprednisolone pulse therapy. The PSs were estimated with logistic regression. PSM was performed using the nearest neighbor method with a 1:1 ratio and a caliper of 0.2. Continuous variables are presented as the median (interquartile range [IQR]), and their differences were compared by Mann‒Whitney tests since all of them did not follow a normal distribution. The changes in ICP and CSF examination results in the two groups before and after treatment were analyzed with the Wilcoxon signed-rank test. The differences in ICP and CSF protein levels before and after treatment were calculated and compared with the Wilcoxon rank-sum test. Categorical variables are expressed as the number and percentage, and comparisons between groups were performed using Fisher’s exact test or the chi-square test as appropriate. Multivariate logistic regression was used to estimate the association between intrathecal treatment and outcome at discharge for the patients with NPSLE. The classification of the outcome at discharge was described previously. Variables unbalanced at baseline or assumed to be clinically significant were entered into the multivariate logistic regression model, and the results are presented as odds ratios (ORs) with 95% confidence intervals (CIs). The endpoint was defined as death from all causes or the first episode of NPSLE recurrence that led to hospitalization in the follow-up. The follow-up period was identified as the time of enrollment until the time of death or the relapse of NPSLE or the last follow-up visit. Survival analysis was performed using the Kaplan‒Meier method, and survival was calculated by the log-rank test in both the unmatched and matched cohorts, with subgroup analysis being further conducted in the matched cohort. Considering the unspecificity of headache symptom, to examine the stability of the results, we performed sensitivity analyses, in which the cases only presented as headache subtype were excluded. PSM was also performed as mentioned previously (using the nearest neighbor method with a 1:1 ratio and a caliper of 0.2 and covariates included sex, age, SLEDAI-2K scores, psychosis, and methylprednisolone pulse therapy) and did survival analysis with the Kaplan‒Meier method in this cohort before and after PSM. Cox regression analysis was used to examine the risk factors for death or the relapse of NPSLE, and the results are presented as hazard ratios (HRs) with 95% CIs. Those variables with clinical significance were entered into the multivariable Cox regression model analysis. In the descriptive statistics, the proportions of missing values in the variables involved were less than 10%, and therefore, the missing values were omitted in this part. No missing values were detected in our multivariate logistic regression and multivariable Cox regression. A two-sided *P* value less than .05 was considered statistically significant. Statistical analyses were performed using R software version 4.0.2.

## Results

### Clinical characteristics of patients with NPSLE

#### Unmatched cohort

**Fig. 1:**
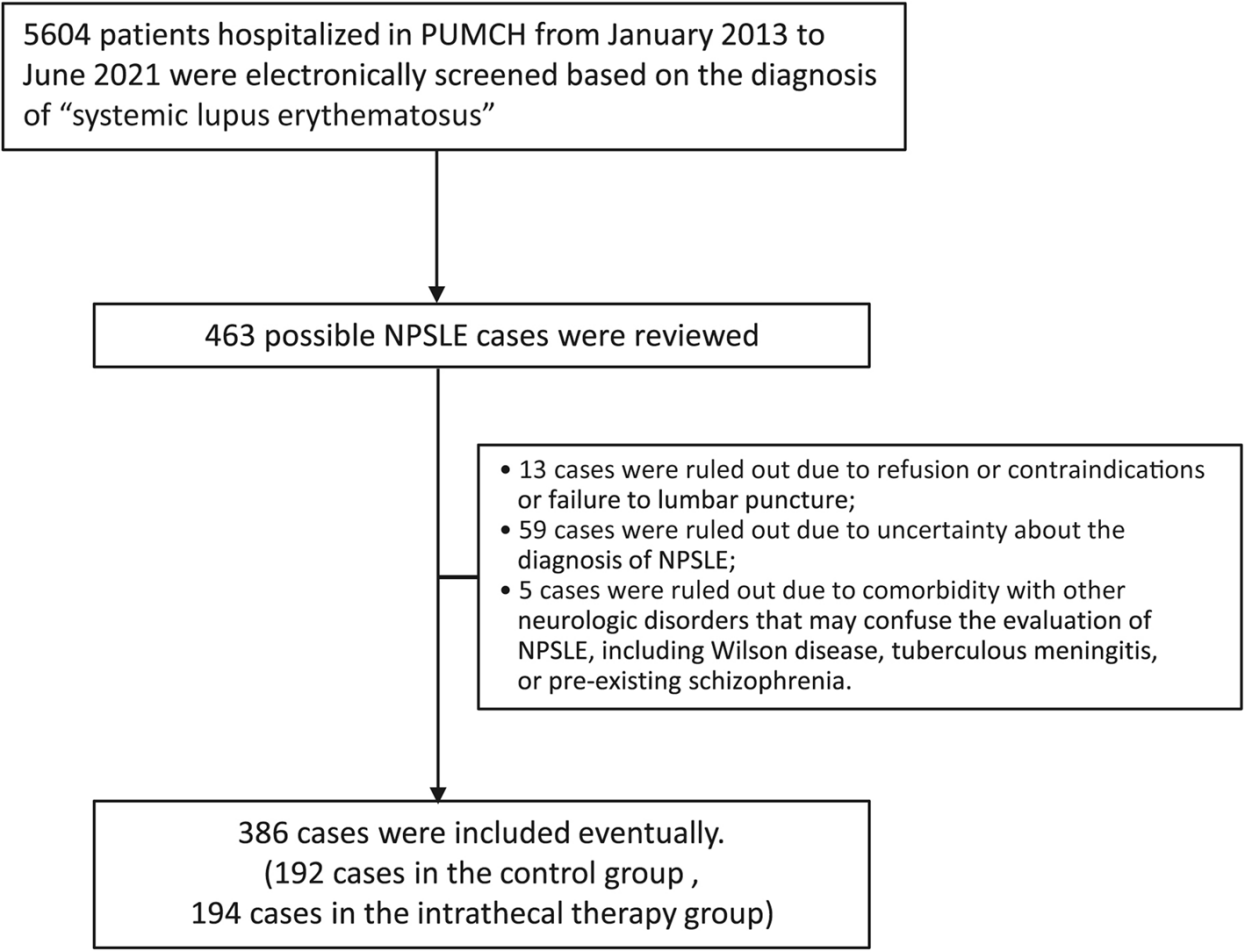
Flowchart of screening of NPSLE patients in this study. PUMCH: Peking Union Medical College Hospital; NPSLE: neuropsychiatric systemic lupus erythematosus

Figure 1 shows the flow diagram for the inclusion of the patients in the final study cohort. Of the 386 NPSLE patients, 194 received intrathecal treatment. All the patients involved in this study received standard treatment for SLE. The clinical characteristics and laboratory test results of these patients are shown in Table 1. The baseline SLEDAI-2K score of the intrathecal therapy group was higher than that of the control group (median 17 vs. 14 points, IQR 12–22 vs. 10–19 points, *P* < 0.001). In contrast, higher frequencies of lupus nephritis (62.5% vs. 47.9%, *P* = 0.006) and antiphospholipid syndrome (10.9% vs. 4.12%,* P*=0.019) were shown in the control group than in the intrathecal treatment group.

**Table 1:** Basic characteristics of NPSLE patients included before and after PSM

	**Before PSM**			**After PSM**		
	Control group (*n* = 192)	Intrathecal treatment group (*n* = 194)	*P *value	Control group (*n* = 147)	Intrathecal treatment group (*n* = 147)	*P* value
Age (years, median [IQR])	29[21.75, 40.25]	31[24.00, 39.75]	0.265	29.0[22.5;40.5]	30.0[23.0;40.0]	0.613
Sex (Male%)	26(13.5)	18(9.3)	0.247	14 (9.52)	16 (10.9)	0.847
Duration of SLE (months, median [IQR])	24.00[4.75, 75.00]	24.00[6.00, 96.00]	0.17	24.0[4.50;84.0]	36.0[6.00;96.0]	0.126
System involvement of SLE, *n* (%)						
Fever	111(57.8)	108(55.7)	0.747	81 (55.1)	82 (55.8)	1
Mucocutaneous involvement	113(58.9)	121(62.4)	0.547	84 (57.1)	93 (63.3)	0.340
Musculoskeletal involvement	96(50.0)	101(52.1)	0.762	81 (55.1)	66 (44.9)	0.102
Pulmonary involvement	16(8.3)	21(10.8)	0.51	15 (10.2)	18 (12.2)	0.712
Cardiovascular involvement	47(24.5)	48(24.7)	1	39 (26.5)	32 (21.8)	0.414
Hematological involvement	108(56.2)	120(61.9)	0.309	85 (57.8)	91 (61.9)	0.552
Lupus nephritis	120(62.5)	93(47.9)	0.006*	95 (64.6)	71 (48.3)	0.007*
Serositis	37(19.3)	24(12.4)	0.086	30 (20.4)	22 (15.0)	0.285
Antiphospholipid syndrome	21 (10.9)	8 (4.12)	0.019*	17(11.6)	6(4.08)	0.030*
Lab tests						
Positive anti-dsDNA antibody, *n* (%)	112(58.3)	119(61.3)	0.618	96 (65.3)	89 (60.5%)	0.469
Positive anti-rRNP antibody, *n* (%)	54(28.1)	65(33.5)	0.301	27 (18.4)	34 (23.1%)	0.388
C3 (g/L, median [IQR])	0.53[0.36, 0.73]	0.50[0.31, 0.71]	0.414	0.51[0.34;0.70]	0.50[0.33;0.76]	0.715
C4 (g/L, median [IQR])	0.08[0.04, 0.14]	0.09[0.04, 0.13]	0.972	0.07[0.04;0.13]	0.09[0.05;0.13]	0.206
Lupus anticoagulant (median [IQR])	1.02[0.97, 1.16]	1.03[0.98, 1.12]	0.89	1.02[0.96;1.15]	1.03[0.98;1.12]	0.749
Positive Coombs test, *n* (%)	86(44.8)	78(40.2)	0.419	71 (48.3)	60 (40.8)	0.241
Positive anti-β2GP1 antibody, *n* (%)	33(17.8)	27(14.0)	0.377	28 (19.3)	19 (13.0)	0.194
Positive ACL, *n* (%)	34(17.9)	22(11.4)	0.098	29 (19.9)	16 (11.0)	0.052
SLEDAI 2K (median [IQR])	14[10, 19]	17[12, 22]	<0.001*	15.0[11.0;20.0]	16.0[11.0;21.0]	0.196
Onset with NPSLE (%)	14(7.3)	18(9.4)	0.569	11 (7.48)	12 (8.33)	0.959
CSF examinations						
Pressure of CSF (mmH_2_O, median [IQR])	162[130, 210]	173.50[132, 230]	0.103	165 [131;210]	180 [135;230]	0.134
Increased ICP^a^, *n* (%)	70(36.5)	91(46.9)	0.048*	63 (42.9)	74 (50.3)	0.242
WBC of CSF (10^6^/L, median [IQR])	0[0-2]	0[0–2]	0.137	0[0–2]	0[0–2]	0.145
Protein of CSF (g/L, median [IQR])	0.33[0.26, 0.45]	0.44[0.30, 0.66]	<0.001*	0.32[0.26;0.46]	0.44[0.30;0.67]	<0.001
Increased CSF protein ^b^, *n* (%)	51(26.6)	97(50.0)	<0.001*	43 (29.3)	74 (50.3)	<0.001*
Abnormal cytology, *n* (%)	12(6.4)	24(12.9)	0.05	21 (33.3)	18 (32.7)	1
Methylprednisolone pulse therapy, *n* (%)	95(49.5)	139(71.6)	<0.001*	91 (61.9)	94 (63.9)	0.809

*PSM* Propensity score matching, *β2GP1* β2-Glycoprotein 1, *ACL*, Anticardiolipin antibody, *ICP* Intracranial pressure, *SLEDAI-2K* Systemic Lupus Erythematosus Disease Activity Index 2000 scores

^a^Increased ICP refers to ICP that more than 180 mmH_2_O

^b^Increased CSF protein refers to a level of CSF protein more than 0.5g/L. Significant *P* values are noted with asterisks

There were 17 subtypes of NPSLE in these patients (Supplementary Table 1). The intrathecal treatment group showed a higher percentage of psychosis (29.4% vs. 17.2%, *P* = 0.009), while the control group showed a higher percentage of headache (32.3% vs. 22.7%, *P* = 0.045). More patients in the intrathecal treatment group than in the control group had more than one subtype of NPSLE (52.1% vs. 38.0%, *P* = 0.008). Apart from the systemic evaluation and discussion of each case to make sure that the neuropsychiatric (NP) events could be attributed to SLE, we also calculated the score of the Italian attribution model in the NPSLE patients manifested only one subtype of NPSLE and found that all of them reached the cutoff value of 6 points to attribute the NP events to SLE[17].

Regarding the laboratory results, lumbar puncture at baseline revealed that more patients in the intrathecal treatment group had increased ICP (> 180 mmH_2_O) (46.9% vs. 36.5%,* P* = 0.048) than the control group. In addition, the intrathecal treatment group had a higher level of CSF total protein (median 0.44 vs. 0.33 g/L, IQR 0.30–0.66 vs. 0.26–0.45 g/L, *P* < 0.001) and a higher proportion of patients with increased total protein in CSF (defined by more than 0.5 g/L, 50.0% vs. 26.6%, *P* < 0.001) than the control group. With respect to treatment, steroid pulse therapy was administered to more patients in the intrathecal group than in the control group (71.6% vs. 49.5%, *P* < 0.001). For immunosuppressive agents, CYC was the most common used drug (70.1% in the intrathecal treatment group vs. 59.9% in the control group, *P* = 0.559), but there was no significant difference between the two groups, followed by mycophenolate mofetil (17.5% in the intrathecal treatment group vs. 21.8% in the control group, *P* = 0.374) (Table 2).

**Table 2:** Comparison of the treatment between the control group and the intrathecal treatment group. Significant *P* values are noted with asterisks

	Control group (*n*=192)	Intrathecal treatment group (*n*=194)	*P* value
Immunosuppressors during hospitalization, *n* (%)			
Cyclophosphamide	115(59.9)	136(70.1)	0.559
Mycophenolate Mofetil	42(21.8)	34(17.5)	0.374
Calcineurin inhibitors	10(5.21)	9(4.64)	0.819
Other immunosuppressive agents	3(1.56)	4(2.06)	1
Rituximab	1(0.52)	2(1.03)	1
Combination of immunosuppressive agents	9(4.69)	6(3.10)	0.584
None	12(6.25)	3(1.55)	0.018*
Time of hospitalization (days, median [IQR])	24[16.8,30]	31[20.36]	0.265
Time of follow-up (years, median [IQR])	2.51[1.08,3.80]	2.63[1.18, 4.48]	0.202

#### Propensity score-matched groups

PSM resulted in 147 pairs of patients receiving intrathecal treatment or not, whose characteristics are presented in Table 1. As shown in Supplementary Figure 1, the imbalance of the characteristics between the two groups was minimized after PSM compared with the unmatched cohort, and the absolute standardized difference was less than 0.1 in all variables used for PSM.

### Intrathecal treatment in patients with NPSLE

In total, 508 intrathecal injections were performed on 194 NPSLE patients (Supplementary Figure 2). Most patients received intrathecal injections one to three times (15% one time, 27.3% two times, 42.2% three times). A total of 42.2% (82/194) of patients received intrathecal therapy less than three times, primarily due to decreased ICP (35/82) or a lack of need for intrathecal treatment due to recovery. Most patients (88.6%) received MTX and DEX as described previously[8, 9], while the others received DEX alone owing to the unavailability of MTX over a short time or concern for renal insufficiency.

### The outcomes of patients with NPSLE and identification of risk factors

#### Patients receiving intrathecal therapy had better short-term outcomes at discharge

**Fig. 2:**
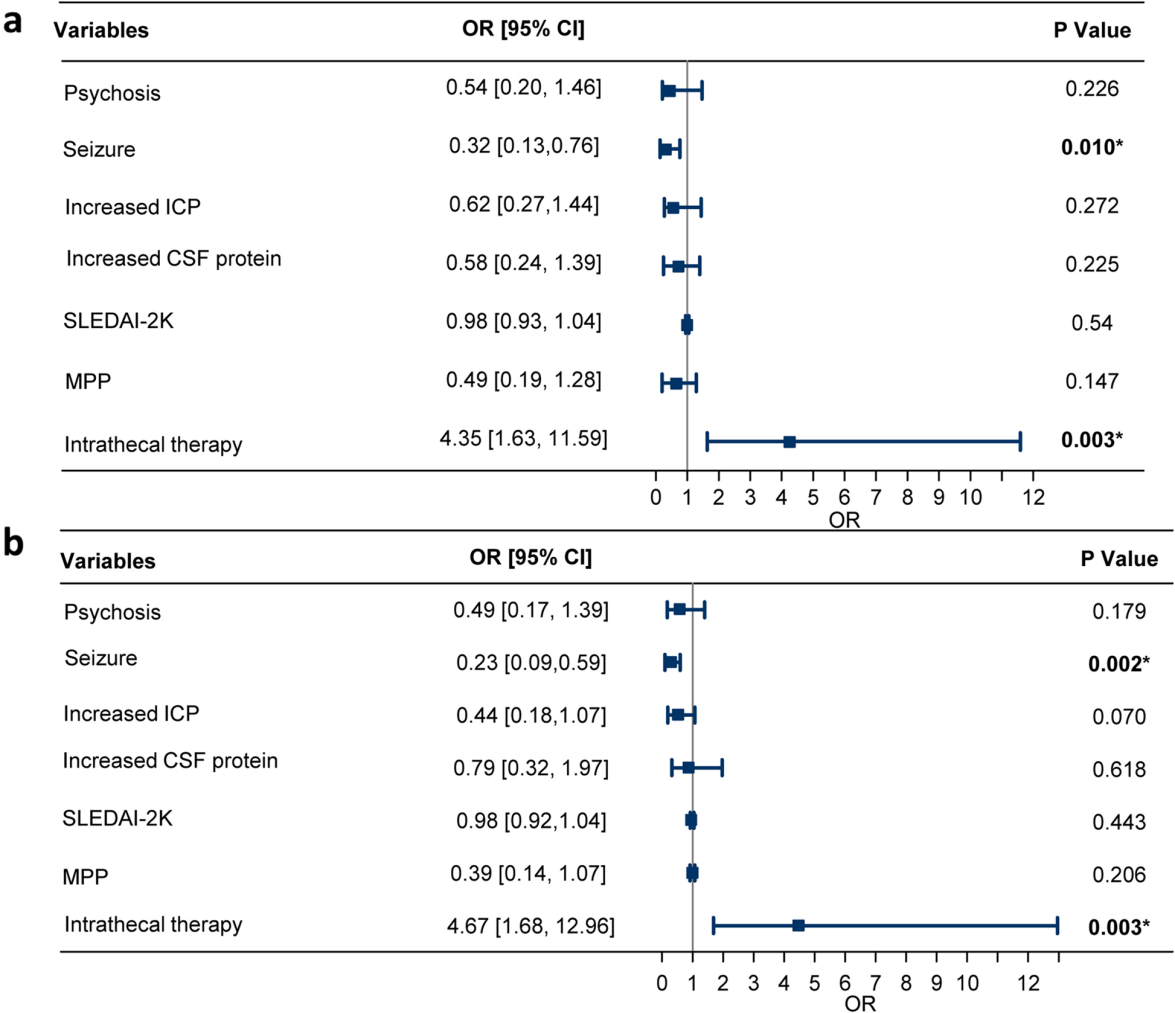
The forest plots of short-term outcome in NPSLE by multivariate logistic regression. The ORs and 95% CIs for complete recovery (**a**) and partial improvement (**b**) in patients in NPSLE. The outcome of exacerbation or death was set as the baseline outcome. SLEDAI-2K: systemic lupus erythematosus disease activity index 2000; MPP: methylprednisolone pulse; NPSLE: neuropsychiatric systemic lupus erythematosus; Increased ICP refers to an ICP that more than 180 mmH_2_O. Increased CSF protein refers to a level of CSF protein more than 0.5g/L. Significant *P* values are noted with asterisks. OR: odds ratio; CI: confidence interval

Multivariate logistic regression was employed in the unmatched cohort, where exacerbation was set as a baseline outcome in this model (Fig. 2). Intrathecal treatment was associated with a favorable outcome of NPSLE, with an OR of 4.35 (95% CI 1.63–11.59,* P* = 0.003) for complete recovery and an OR of 4.67 (95% CI 1.68–12.96,* P* = 0.003) for partial improvement. Patients with seizure disorders were associated with a lower probability of complete recovery (OR 0.32, 95% CI 0.13–0.76, *P* = 0.010) and partial improvement (OR 0.23, 95% CI 0.09–0.59, *P* = 0.002). All the other variables (psychosis, SLEDAI-2K score, increased ICP, increased CSF protein, and methylprednisolone pulse) in the multivariate logistic model were not associated with outcome at discharge.

#### Intrathecal treatment decreased ICP and total protein in CSF to a greater extent than the control treatment

Next, we investigated the changes in CSF examination results before and after intrathecal treatment, since we administered three intrathecal injections to most patients as the experience of our center. Before and after treatment, CSF examination results were collected (165/194 patients in the intrathecal treatment group and 29/192 patients in the control group). Compared with the control group, the intrathecal treatment group demonstrated a more significant decline in ICP (*P* < 0.001) and levels of total CSF protein (*P* < 0.001) compared with baseline values (Supplementary Figure 3).

#### Intrathecal treatment improved the long-term prognosis of NPSLE

**Fig. 3:**
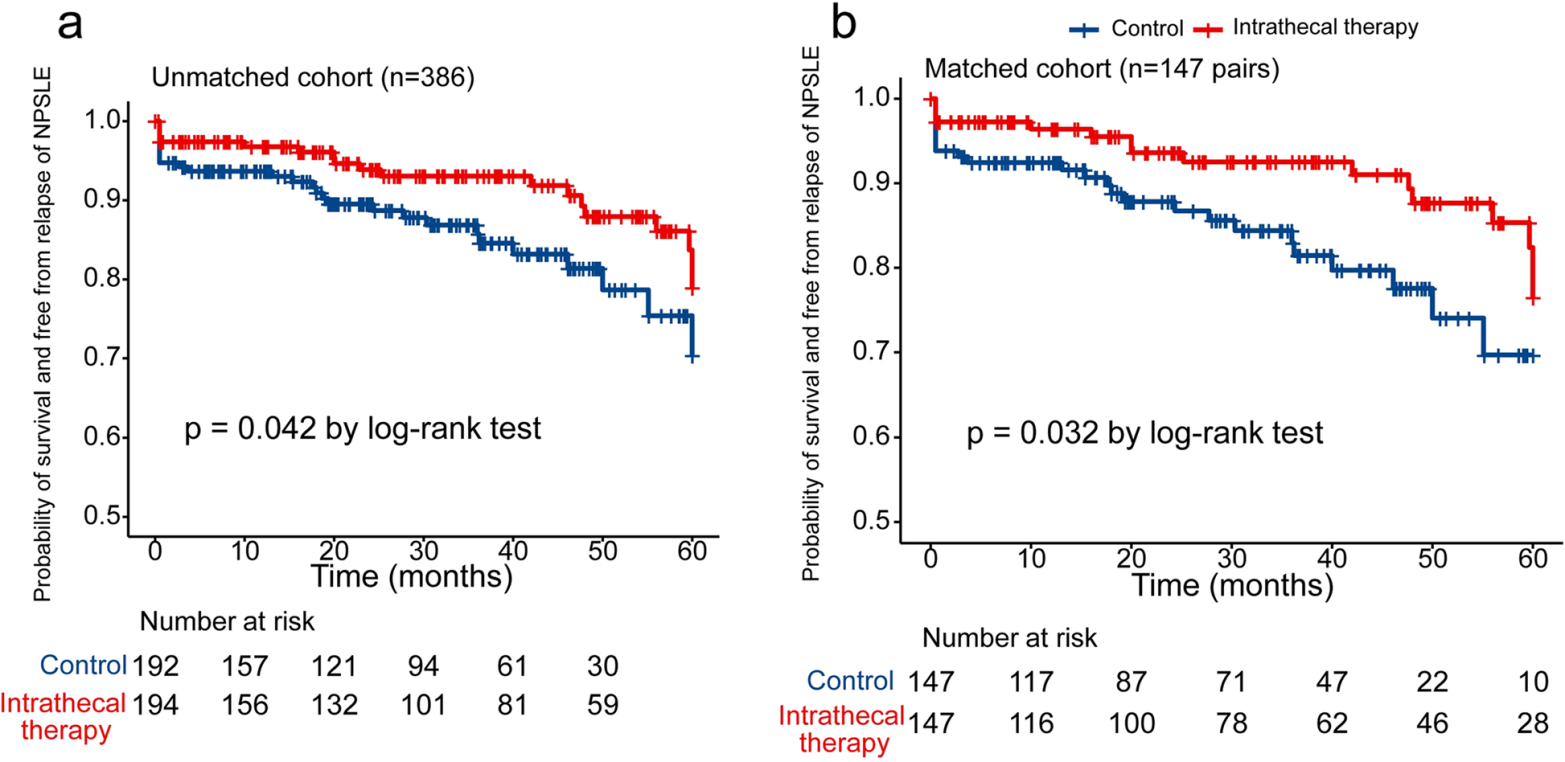
The survival analysis plot of NPSLE patients during follow-up. **a** Kaplan–Meier plot of patients with NPSLE in the intrathecal treatment group or the control group in the unmatched cohort. **b** Kaplan–Meier plot of patients with NPSLE in the intrathecal treatment group or the control group in the matched cohort by propensity score matching. Note: NPSLE: neuropsychiatric systemic lupus erythematosus

Furthermore, we investigated the influence of intrathecal treatment on the long-term prognosis of NPSLE. The endpoint event was defined as death from all causes or a relapse of NPSLE causing hospitalization. The overall follow-up time for the 386 patients was 1007.69 person-years. The median durations of follow-up in the intrathecal treatment group and the control group were 2.63 years (IQR 1.18–4.48 years) and 2.51 years (IQR 1.08–3.80 years), respectively (Table 2). In total, 47 endpoint events were recorded, where 28 patients experienced a relapse of NPSLE and 19 patients died. The main causes of death were SLE-related causes (13 patients; 68.4%) and infection (6 patients; 31.6%). In the survival analysis, more patients in the intrathecal therapy group were free from the defined endpoint events than patients in the control group both in the unmatched cohort (*P* = 0.042 by log-rank test, Fig. 3a) and in the matched cohort (*P* = 0.032 by log-rank test, Fig. 3b). Considering that headache is a unspecific manifestation of NPSLE, in sensitivity analysis, we excluded the cases that only presented headache symptom (*n*=67). In this cohort, the intrathecal therapy group was still more likely to be free from the defined endpoint events than patients in the control group both in the unmatched cohort (*P* = 0.023 by log-rank test, Supplementary Figure 4a) and in the matched cohort (*P* = 0.018 by log-rank test, Supplementary Figure 4b).In the univariate Cox analysis (Table 3), intrathecal treatment was a protective factor (HR 0.55, 95% CI 0.30–0.99,* P* = 0.045 in the unmatched cohort; HR 0.50, 95% CI 0.27–0.95,* P* = 0.035 in the matched cohort). Details of the univariable Cox analysis in the unmatched and matched cohorts are shown in Table 3. In the final multivariable Cox regression model (variables including intrathecal therapy, increased CSF protein, ACS, methylprednisolone pulse therapy, CYC treatment), intrathecal therapy was found to be an independent protective factor (adjusted HR 0.42, 95% CI 0.22–0.82, *P* =0.0108), while increased CSF protein (adjusted HR 2.01, 95% CI 1.06–3.82, *P* = 0.034) and ACS (adjusted HR 3.75, 95% CI 1.95–7.22, *P* < 0.001) were found to be independent risk factors for a poor prognosis of NPSLE. It is noticeable that methylprednisolone pulse therapy and CYC were also included in the multivariable Cox regression model, and thus, the association of intrathecal treatment with better prognosis persisted after adjusting for confounding factors, including methylprednisolone pulse therapy and CYC treatment (for methylprednisolone pulse therapy, adjusted HR 0.97, 95% CI 0.50–1.86, *P* = 0.922, and for CYC treatment, adjusted HR 0.67, 95% CI 0.36–1.25, *P* = 0.205).

**Table 3:** Univariable Cox regression for predictors of death or relapse of NPSLE in unmatched cohort and matched cohort after PSM

	Univariable Cox analysis in unmatched cohort		Univariable Cox analysis in matched cohort by PSM	
Variables	HR (95%CI)	*P* value^b^	HR (95%CI)	*P* value^b^
Intrathecal therapy	0.55(0.3–0.99)	0.045*	0.50(0.27–0.95)	0.035*
Sex (Male)	0.34(0.08–1.39)	0.133	0.47(0.11–1.93)	0.291
System involvement of SLE				
Mucocutaneous	0.85(0.47–1.52)	0.583	0.90(0.48–1.68)	0.730
Musculoskeletal	0.72(0.4–1.28)	0.265	0.78(0.42–1.44)	0.426
Pulmonary involvement	1.46(0.62–3.43)	0.391	1.42(0.6–3.37)	0.431
Cardiovascular involvement	1.9(1.04–3.48)	0.037*	2.05 (1.07–3.92)	0.031*
Hematological involvement	1.08(0.6–1.94)	0.791	0.92(0.49–1.72)	0.797
Lupus nephritis	1.28(0.71–2.3)	0.415	1.28(0.68–2.42)	0.446
Serositis	2.2(1.16–4.17)	0.016*	2.01 (1.05–3.87)	0.035*
SLEDAI-2K	1.01(0.97–1.04)	0.774	1.00(0.96–1.04)	0.847
Subtype of NPSLE				
Headache	0.5(0.23–1.07)	0.074	0.48 (0.21–1.09)	0.079
Seizure disorder	2.05(1.15–3.66)	0.015*	2.03 (1.1–3.75)	0.024*
Acute confusional state	3.73(2.03–6.87)	<0.001*	3.55 (1.86–6.78)	<0.001*
Cerebrovascular disease	0.69(0.25–1.91)	0.471	0.84(0.3–2.36)	0.742
Mood disorder	1.54(0.69–3.45)	0.295	1.94(0.86–4.38)	0.112
Cognitive dysfunction	0.73(0.29–1.85)	0.507	0.89(0.35–2.28)	0.812
Aseptic meningitis	0.39(0.05–2.86)	0.358	0.94(0.68–3.56)	0.906
Anxiety	0.91(0.13–6.6)	0.924	1.25(0.17–9.08)	0.827
Psychosis	0.71(0.72–4.63)	0.386	0.77(0.34–1.74)	0.528
Myelopathy	1.83(0.72–4.63)	0.203	1.17(0.36–3.8)	0.795
Demyelinating syndrome	1.68(0.52–5.43)	0.385	1.50(0.46–4.86)	0.503
Guillain–Barré syndrome	1.45(0.2–10.54)	0.716	1.27 (0.17–9.27)	0.816
Mononeuropathy	0(0–Inf)	0.997	0(0–Inf)	0.996
Movement disorder	1.3(0.31–5.37)	0.716	1.43(0.35–5.95)	0.620
Cranial neuropathy	1.11(0.27–4.58)	0.884	1.39(0.34–5.77)	0.649
Plexopathy	3.86(0.53–28.15)	0.183	3.38(0.46–24.75)	0.231
Polyneuropathy	1.72(0.68–4.35)	0.255	1.50(0.53–4.24)	0.441
Methylprednisolone pulse therapy	1.03(0.57–1.86)	0.922	0.96 (0.51–1.81)	0.911
CYC use	0.57 (0.32–1.00)	0.051	0.63 (0.34–1.16)	0.137
Increased ICP^a^	0.91(0.51–1.62)	0.753	0.78(0.42–1.46)	0.440
Increased levels of protein in CSF^b^	1.39(0.78–2.41)	0.258	1.47(0.8–2.72)	0.216
Abnormal CSF cytology	1.13(0.45–2.87)	0.793	1.17(0.41–3.3)	0.771

*β2GP1* β2-Glycoprotein 1, *ACL* Anticardiolipin antibody, *ICP* Intracranial pressure, *SLEDAI-2K* Systemic Lupus Erythematosus Disease Activity Index 2000 scores

^a^Increased ICP refers to ICP that more than 180mmH_2_O

^b^Increased levels of protein in CSF refers to a level that more than 0.5g/L of CSF protein. Significant *P* values are noted with asterisks

#### Subgroup analyses identified potential target population of NPSLE patients for intrathecal treatment

**Fig. 4:**
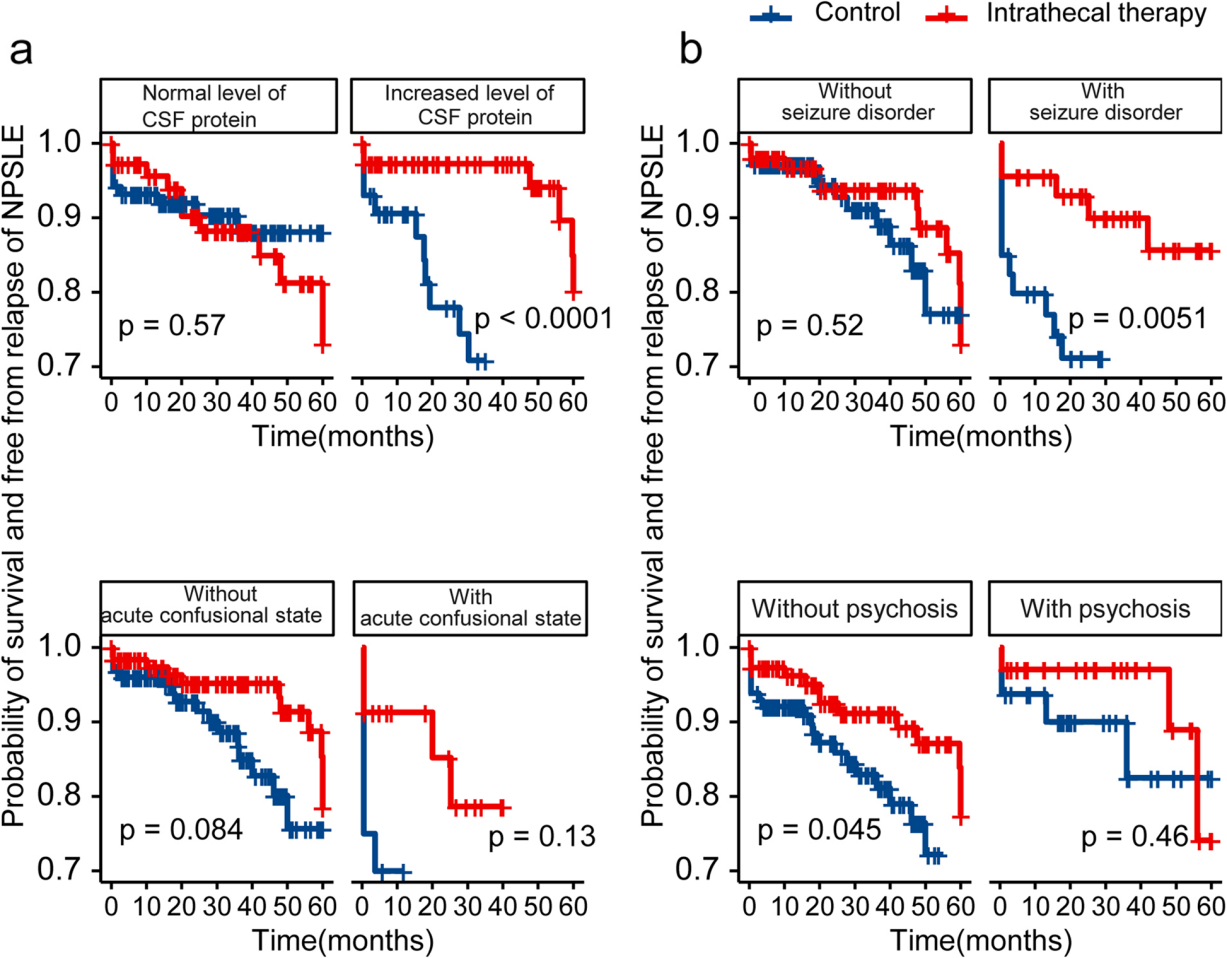
Subgroup survival analysis in the matched cohort. **a** The subgroup Kaplan–Meier analysis dependent on the CSF protein levels (top) and the existence of acute confusional state (bottom). **b** The subgroup Kaplan–Meier analysis dependent on the existence of seizure disorders (top) and psychosis (bottom)

To explore the potential target population of NPSLE patients for intrathecal treatment, subgroup survival analyses were performed in the matched cohort after PSM. As expected, NPSLE patients with increased levels of CSF protein acquired an outstanding outcome benefit from intrathecal treatment compared with control group patients (*P* < 0.001 by log-rank test), while those with normal levels of CSF protein did not obtain a significant outcome benefit (*P* = 0.57 by log-rank test). (Fig. 4a, top). We also explored the prognosis of different NPSLE subtypes in the subgroup survival analysis, including seizure disorder, ACS, psychosis, and headache (Fig. 4a, b, Supplementary Figure 5). Intrathecal treatment seemed to have a positive influence in subgroups divided by some subtypes of NPSLE, but the limited sample size in each group constrained us from reaching a conclusion with statistical meaning in most situations.

### The safety of intrathecal treatment for NPSLE

After intrathecal treatment, low ICP (lower than 80 mmH_2_O or headache attributed to intracranial hypotension) was observed in 18% of patients receiving intrathecal treatment. In the control group, 3.12% of patients had a reversible headache after lumbar puncture, which was attributed to intracranial hypotension after lumbar puncture. In addition, in the intrathecal treatment group, two patients reported temporary unilateral lower limb numbness, which recovered in a few hours. One patient reported a transient wheal on the trunk. No secondary CNS infection was observed in the intrathecal treatment group during hospitalization and the follow-up periods.

## Discussion

In this large, single-center cohort of patients hospitalized for NPSLE, we found that intrathecal injection of MTX and DEX was associated with a better prognosis of NPSLE in the overall population and in the matched population after balancing the confounding influence from different subtypes and treatment intensities. Apart from the clinical events, intrathecal treatment with MTX and DEX could also reduce the CSF pressure and protein levels to a larger extent compared with the control treatment, indicating its local anti-inflammatory effects. We also found that patients with increased levels of CSF protein (more than 0.5 g/L) at baseline were more likely to benefit from intrathecal therapy.

Neuropsychiatric involvement, as one of the most severe complications of SLE, is a major contributor to mortality in SLE, surpassed only by lupus nephritis[18]. Clinical manifestations of NPSLE defined by ACR in 1999[3] include ACS, seizure disorders, psychosis, aseptic meningitis, myelopathy, Guillain–Barré syndrome, mononeuropathy, polyneuropathy, cranial neuropathy, polyneuropathy, plexopathy, movement disorder, cerebrovascular disease, mood disorder, anxiety disorder, cognitive dysfunction, headache, myasthenia gravis, and autonomic disorder. For the management of NPSLE, according to the 2010 EULAR recommendation for the management of NPSLE[5], general treatment includes the control of aggravating factors (such as blood pressure and metabolic factors) and symptomatic treatment including antipsychotics and anti-epileptics as needed. Disease-specific therapy depends on the underlying process of NPSLE. For NPSLE attributed to antiphospholipid antibodies, anticoagulation or antiplatelet treatment serves as the cornerstone of treatment. In NPSLE reflecting autoimmune-mediated neuroinflammation and in the presence of lupus activity, immunosuppressive therapy with high-dose corticosteroids alone or combined with CYC or azathioprine is recommended. However, only oral glucocorticoids and intravenous CYC have been tested in clinical trials and revealed positive outcomes[19, 20]. Specifically, in the controlled clinical trial of CYC in NPSLE, the group treated with intravenous CYC combined with glucocorticoids (*n*=19, response rate 95%) showed a higher response rate than the group treated with glucocorticoids alone (*n*=12, response rate 46%). Other treatments, including rituximab (combined with glucocorticoids, reported response rate: 73–100%)[21–23] and plasma exchange (combined with glucocorticoids and immunosuppressants, reported response rate: 74–100%)[24, 25], have also been explored in severe NPSLE refractory to standardized immunosuppressive therapy.

Although the mortality of NPSLE has decreased recently due to progress in diagnosis and treatment, unmet needs are still outstanding in this field[26]. For example, most controlled clinical trials of new therapeutic agents for SLE have excluded SLE patients with severe neuropsychiatric manifestations. Such a gap has made it difficult for NPSLE patients to access newly developed drugs for SLE. In this case, we aimed to reintroduce intrathecal treatment as a therapeutic choice for patients with NPSLE, which reflects an immune-mediated inflammatory process in the nervous system, especially for the severe or refractory cases. However, it is not a common therapy for NPSLE, partially because of the limited evidence provided by previous studies. Intrathecal injection of MTX and DEX for NPSLE, adapted from the intrathecal therapy for leukemia with CNS involvement, is expected to reach a higher local drug concentration in the CNS, thus enhancing its therapeutic effect on NPSLE in a more direct way. Previous studies of intrathecal treatment for NPSLE patients unanimously exhibited a positive outcome at discharge, but none of them included follow-up information on NPSLE patients treated by intrathecal injection[8, 9, 11]. Moreover, some significant points about intrathecal treatment are still unknown, including its influence on CSF measurements, such as protein levels, its long-term influence on the prognosis of patients, its long-term safety, and the optimal NPSLE subgroup for intrathecal treatment. Intrathecal therapy has been used at PUMCH for nearly 30 years. To our knowledge, our study included the largest cohort of NPSLE patients receiving intrathecal therapy and proved that intrathecal treatment was a protective factor for NPSLE from a series of aspects. For the short-term effect, evaluated by the outcome at discharge, intrathecal injection of MTX and DEX was associated with favorable outcomes, with both complete recovery and partial improvement, compared with the control treatment, in accordance with previous studies. Intrathecal treatment for NPSLE patients also improved long-term prognosis, as evidenced by the multivariable Cox regression model. The exploratory analysis found that NPSLE patients with increased levels of CSF protein were more likely to benefit from intrathecal treatment. We also investigated the safety of intrathecal treatment and found that asymptomatic low ICP was relatively common, and there were no severe adverse effects in our patients, indicating the good tolerability and safety of intrathecal treatment.

Since the present study was retrospective, it is important to reduce all potential biases. Therefore, efforts were made to minimize the potential biases to draw more reliable conclusions. Above all, in the screening process of the study, every case was systemically evaluated, and patients with neuropsychiatric events that may be attributed to secondary causes other than NPSLE were excluded. In addition, since the decision to perform intrathecal treatment was made by attending physicians after informed consent, the allocation of NPSLE patients to the intrathecal treatment group or not was not a random process. It was understandable that the patients in the intrathecal treatment group had a more severe clinical condition (higher SLEDAI-2K score, higher proportion of more than one subtype of NPSLE in the same patient) and had higher proportions receiving steroid pulse treatment. To balance the different conditions in the control group and the intrathecal treatment group, we applied PSM methodology to construct a comparable cohort. The patients with NPSLE in the matched cohort also had a better prognosis in the Kaplan‒Meier analysis and in the univariable Cox analysis. In addition to PSM, multivariable regression, including logistic and Cox regression, was performed to investigate the association of intrathecal therapy with outcome after adjusting for potential confounding factors. In summary, after balancing the variance in disease condition and treatment intensity, intrathecal treatment was associated with a better prognosis of NPSLE. Furthermore, given the great heterogeneity in NPSLE (19 subtypes), we explored the subpopulation of NPSLE that was better for intrathecal treatment. A statistically significant conclusion was barely reached in the subgroup based on the subtype of NPSLE owing to the small sample size of a specific NPSLE subtype. However, when NPSLE patients were divided into two subgroups according to CSF protein levels, those with increased levels of CSF protein were more likely to acquire benefits from intrathecal treatment, while in the subgroup with normal levels, there was no difference between those who received intrathecal treatment and those who did not. Considering that elevated protein levels in CSF reflect damage to the blood‒brain barrier and intrathecal inflammation, the results were reasonable, indicating that intrathecal treatment could become a useful supplementary treatment for those with obvious intrathecal inflammation. Notably, we also compared ICP and CSF protein levels at baseline and after treatment, and the extent of the decrease in ICP and CSF protein levels was larger in the intrathecal treatment group than in the control group, suggesting more alleviation of the intrathecal inflammatory process by intrathecal treatment.

Our study has several limitations. First, although we adjusted for the confounders through the application of PSM methodology, the retrospective nature of this study inherently introduces unavoidable selection biases. Further prospective cohort studies should be performed in the future to obtain more solid evidence. Second, this study was based on our single-center experience, and further validation in external cohorts in prospective studies is needed. Last but not least, the evaluation of NPSLE outcomes in the retrospective study was relatively vague based on the global assessment of rheumatologists, and more detailed evaluation of the outcomes of NPSLE may help in future studies.

Based on this study and the long-term experience at our center, although intrathecal treatment is an uncommon therapy for NPSLE, it could achieve higher drug concentrations in the CNS, enhanced the effects of conventional treatment, and promoted rapid control of NPSLE. In addition, intrathecal treatment was especially beneficial for patients with infection, which might limit the effects of aggressive systemic treatments such as methylprednisolone pulse therapy. Moreover, our data also showed that intrathecal treatment was safe and well tolerated, both in short- and long-term observations.

## Conclusions

In conclusion, we conducted a retrospective study and investigated the efficacy and safety of intrathecal injection of MTX and DEX in patients with NPSLE. Intrathecal treatment was a protective factor for patients with NPSLE both on a short-term and long-term basis and may serve as a valuable additional therapy for NPSLE patients, especially for those with elevated CSF protein levels.

## Supplementary Information

**Additional file 1: Supplementary Table 1.** Subtypes of NPSLE in the two groups before and after PSM. **Supplement Figure 1.** The distribution of variables before PSM and after PSM. **Supplementary Figure 2.** The treemap of times and composition of drugs for intrathecal treatment of NPSLE patients. a. Q-Q plots of each covariate from 2 groups before and after PSM. b. Histogram of propensity score in the intrathecal treatment group (the upper row) and the control group (the bottom row) before and after PSM. c. Standardized means differences of the covariates included in PSM (MPP, SLEDAI-2K, psychosis, headache, sex, age) before and after PSM.d. Scatter plot of propensity scores before and after PSM in the intrathecal treatment group (treated units) and the control group (control units). Note: PSM, propensity scores matching, MPP, methylprednisolone pulse; SLEDAI-2K, Systemic Lupus Erythematosus Disease Activity Index 2000. **Supplementary Figure 3.** The comparison of changes of CSF protein and intracranial pressure after treatment between the intrathecal treatment group and the control group. a. the changes of CSF protein after treatment between the patients with NPSLE received intrathecal treatment (*n*=165) and those who did not (*n*=29). b. the changes of intrathecal treatment after treatment between the patients with NPSLE received intrathecal treatment (*n*=165) and those who did not (*n*=29). Group differences were assessed using Wilcoxon signed rank test. Note: CSF: craniospinal fluid. **Supplementary Figure 4.** The Kaplan-Meier plot of NPSLE patients during follow-up after the cases only presented headache were excluded (*n*=69). a. Kaplan-Meier plot of patients with NPSLE in the intrathecal treatment group or the control group in the unmatched cohort. b. Kaplan-Meier plot of patients with NPSLE in the intrathecal treatment group and the control group in the matched cohort by propensity score matching (PSM); The covariates used in PSM included sex, age, methylprednisolone pulse, SLEDAI-2K scores, psychosis. Note: NPSLE: neuropsychiatric systemic lupus erythematosus. **Supplementary Figure 5.** The Kaplan-Meier analysis plot of NPSLE patients only presented headache symptom in the two groups(*n*=67). Note: NPSLE: neuropsychiatric systemic lupus erythematosus.

## Data Availability

All data generated or analyzed during this study are included in this published article [and its supplementary information files].

## Abbreviations

SLE: Systemic lupus erythematosus
NPSLE: Neuropsychiatric systemic lupus erythematosus
PSM: Propensity score matching
MTX: Methotrexate
DEX: Dexamethasone
CYC: Cyclophosphamide
ACR: American College of Rheumatology
EULAR: European League Against Rheumatism
IHS: International Headache Society
SLICC: Systemic Lupus International Collaborating Clinics
PUMCH: Peking Union Medical College Hospital
SLEDAI-2K: Systemic Lupus Erythematosus Disease Activity Index 2000
BILAG: British Isles Lupus Assessment Group
ICP: Intracranial pressure
CSF: Cerebrospinal fluid
OR: Odds ratio
HR: Hazard ratio
CI: Confidence interval
IQR: Interquartile range
