# Correction: The influence of intrathecal injection of methotrexate and dexamethasone on neuropsychiatric systemic lupus erythematosus (NPSLE): a retrospective cohort study of 386 patients with NPSLE

**DOI:** 10.1186/s13075-023-03056-0

**Published:** 2023-04-29

**Authors:** Yuxue Nie, Boyuan Sun, Xin He, Minmin Zheng, Di Wu, Yunjiao Yang, Li Zhang, Wei Bai, Nan Jiang, Lin Qiao, Can Huang, Shuang Zhou, Jiaxin Zhou, Linyi Peng, Jingwen Niu, Mengtao Li, Yan Zhao, Xiaofeng Zeng, Li Wang, Wen Zhang

**Affiliations:** 1 Department of Rheumatology, National Clinical Research Center for Dermatologic and Immunologic Diseases, State Key Laboratory of Complex Severe and Rare Diseases, Key Laboratory of Rheumatology and Clinical Immunology, Ministry of Education, Peking Union Medical College Hospital, Chinese Academy of Medical Science and Peking Union Medical College, Beijing, China; 2 Department of Hematology and Rheumatology, Zhongshan Boai Hospital Affiliated to Southern Medical University, Zhongshan, China; 3 Department of Neurology, Peking Union Medical College Hospital, Chinese Academy of Medical Sciences, Beijing, China

**Correction: Arthritis Res Ther 25, 50 (2023)**

**https://doi.org/10.1186/s13075-023-03030-w**

Following publication of the original article [1], the authors reported an error in the section “Intrathecal therapy” of Methods (Page 3 of 14, Para 5, Line 8) that “5 ml” should be “10 ml”.

The original article has been corrected.
