# Supplementary material for: The influence of intrathecal injection of methotrexate and dexamethasone on neuropsychiatric systemic lupus erythematosus (NPSLE): a retrospective cohort study of 386 patients with NPSLE
Source: Arthritis Res Ther. 2023 Mar 28;25:50. doi: 10.1186/s13075-023-03030-w (PMC10045150; doi:10.1186/s13075-023-03030-w)
Supplement: Supplementary file 1 — Additional file 1: Supplementary Table 1. Subtypes of NPSLE in the two groups before and after PSM. Supplement Figure 1. The distribution of variables before PSM and after PSM. Supplementary Figure 2. The treemap of times and composition of drugs for intrathecal treatment of NPSLE patients. a. Q-Q plots of each covariate from 2 groups before and after PSM. b. Histogram of propensity score in the intrathecal treatment group (the upper row) and the control group (the bottom row) before and after PSM. c. Standardized means differences of the covariates included in PSM (MPP, SLEDAI-2K, psychosis, headache, sex, age) before and after PSM.d. Scatter plot of propensity scores before and after PSM in the intrathecal treatment group (treated units) and the control group (control units). Note: PSM, propensity scores matching, MPP, methylprednisolone pulse; SLEDAI-2K, Systemic Lupus Erythematosus Disease Activity Index 2000. Supplementary Figure 3. The comparison of changes of CSF protein and intracranial pressure after treatment between the intrathecal treatment group and the control group. a. the changes of CSF protein after treatment between the patients with NPSLE received intrathecal treatment (n=165) and those who did not (n=29). b. the changes of intrathecal treatment after treatment between the patients with NPSLE received intrathecal treatment (n=165) and those who did not (n=29). Group differences were assessed using Wilcoxon signed rank test. Note: CSF: craniospinal fluid. Supplementary Figure 4. The Kaplan-Meier plot of NPSLE patients during follow-up after the cases only presented headache were excluded (n=69). a. Kaplan-Meier plot of patients with NPSLE in the intrathecal treatment group or the control group in the unmatched cohort. b. Kaplan-Meier plot of patients with NPSLE in the intrathecal treatment group and the control group in the matched cohort by propensity score matching (PSM); The covariates used in PSM included sex, age, methylprednisolone pulse, SLE [file 13075_2023_3030_MOESM1_ESM.docx]

Supplementary materials

**Supplementary table 1 Subtypes of NPSLE in the two groups before and after PSM**

|  |  |  |  |  |  |  | |  |
| --- | --- | --- | --- | --- | --- | --- | --- | --- |
|  | **Unmatched cohort** | |  |  | **Matched cohort** | | |  |
| **Characteristics of NPSLE, n (%)** | **Control group (n=192)** | **Intrathecal treatment group (n=194)** | ***P* Value** |  | **Control group (n=147)** | **Intrathecal treatment group (n=147)** | ***P* Value** | |
| Headache | 62(32.3) | 44(22.7) | 0.045* |  | 43 (29.3) | 41 (27.9) | | 0.897 |
| Polyneuropathy | 10(5.2) | 11(5.7) | 1 |  | 7 (4.76) | 8 (5.44) | | 1 |
| Plexopathy | 2(1.0) | 1(0.5) | 0.993 |  | 1 (0.68) | 1 (0.68) | | 1 |
| Cranial neuropathy | 3(1.6) | 10(5.2) | 0.094 |  | 2 (1.36) | 7 (4.76) | | 0.173 |
| Movement disorder | 9(4.7) | 5(2.6) | 0.403 |  | 6 (4.08) | 5 (3.40) | | 1 |
| Mononeuropathy | 4(2.1) | 0(0.0) | 0.129 |  | 1 (0.68) | 0 (0.00) | | 1 |
| Guillain Barré syndrome | 1(0.5) | 3(1.5) | 0.623 |  | 1 (0.68) | 3 (2.04) | | 0.622 |
| Demyelinating syndrome | 4(2.1) | 11(5.7) | 0.119 |  | 4 (2.72) | 10 (6.80) | | 0.171 |
| Psychosis | 33(17.2) | 57(29.4) | 0.007* |  | 32 (21.8) | 34 (23.1) | | 0.889 |
| Myelopathy | 5(2.6) | 14(7.2) | 0.063 |  | 3 (2.04) | 11 (7.48) | | 0.055 |
| Anxiety | 1(0.5) | 6(3.1) | 0.131 |  | 1 (0.68) | 4 (2.72) | | 0.371 |
| Aseptic meningitis | 8(4.2) | 12(6.2) | 0.506 |  | 4 (2.72) | 9 (6.12) | | 0.256 |
| Cognitive dysfunction | 23(12.0) | 33(17.0) | 0.208 |  | 19 (12.9) | 23 (15.6) | | 0.617 |
| Mood disorder | 13(6.8) | 22(11.3) | 0.166 |  | 10 (6.80) | 15 (10.2) | | 0.403 |
| Cerebrovascular disease | 28(14.6) | 21(10.8) | 0.339 |  | 21 (14.3) | 16 (10.9) | | 0.482 |
| Acute confusional state | 24(12.8) | 32(16.5) | 0.376 |  | 20 (13.6) | 23 (15.6) | | 0.741 |
| Seizure disorder | 48(25.1) | 57(29.4) | 0.411 |  | 40 (27.2) | 45 (30.6) | | 0.607 |
| More than one type of presentation | 73(38.0) | 101(52.1) | 0.008* |  |  |  | |  |

Note: For those patients exhibited more than one subtype of NPSLE, corresponding subtypes were recorded and involved into statistical analyses respectively. Note:PSM, propensity score matching. Significant P values are noted with asterisks.

Supplement figure 1 the distribution of variables before PSM and after PSM.


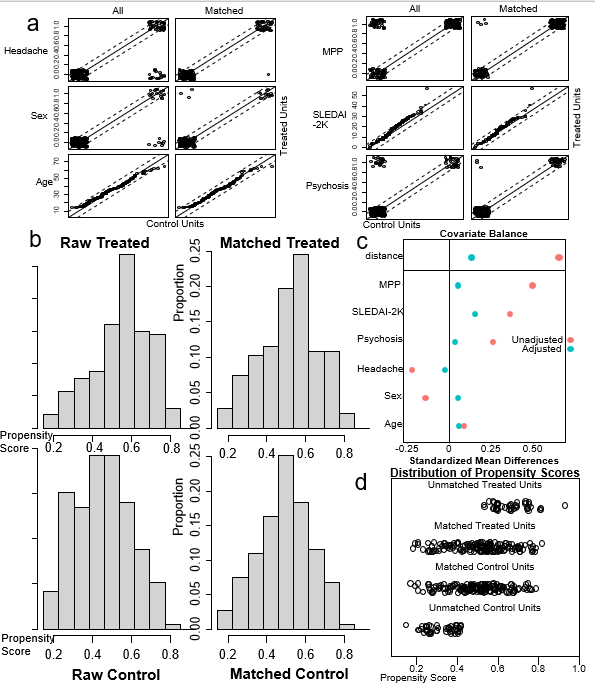


a. Q-Q plots of each covariate from 2 groups before and after PSM. b. Histogram of propensity score in the intrathecal treatment group (the upper row) and the control group (the bottom row) before and after PSM. c. Standardized means differences of the covariates included in PSM (MPP, SLEDAI-2K, psychosis, headache, sex, age) before and after PSM.d. Scatter plot of propensity scores before and after PSM in the intrathecal treatment group (treated units) and the control group (control units). Note: PSM, propensity scores matching, MPP, methylprednisolone pulse; SLEDAI-2K, Systemic Lupus Erythematosus Disease Activity Index 2000

Supplementary figure 2 The treemap of times and composition of drugs for intrathecal treatment of NPSLE patients.


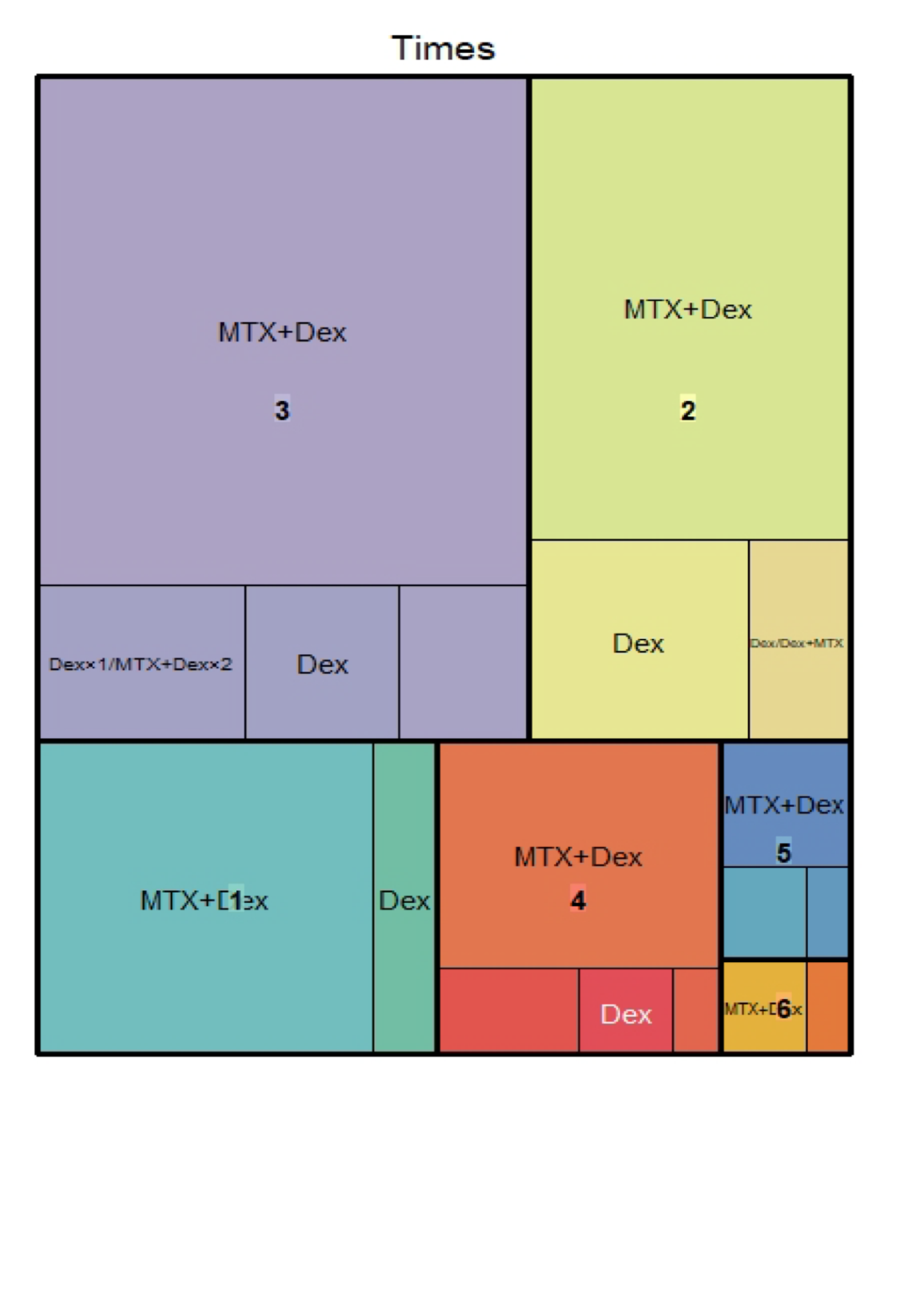


Note: The numbers in the figure indicated the times of intrathecal treatment in 194 patients with NPSLE. MTX: methotrexate; NPSLE: neuropsychiatric lupus erythematosus; Dex: dexamethasone.

Supplementary figure 3


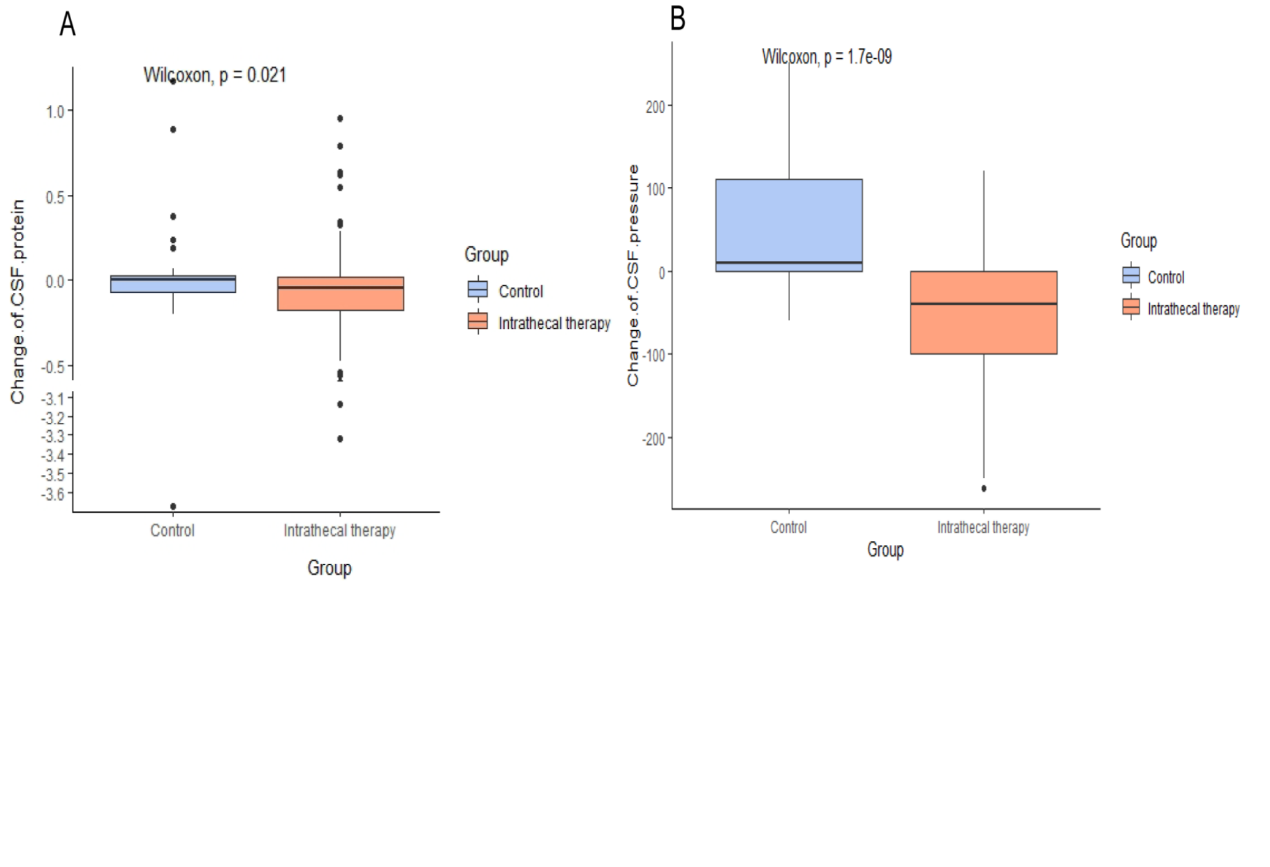


Supplementary figure 3

The comparison of changes of CSF protein and intracranial pressure after treatment between the intrathecal treatment group and the control group. a. the changes of CSF protein after treatment between the patients with NPSLE received intrathecal treatment (n=165) and those who did not (n=29). b. the changes of intrathecal treatment after treatment between the patients with NPSLE received intrathecal treatment (n=165) and those who did not (n=29). Group differences were assessed using Wilcoxon signed rank test. Note: CSF: craniospinal fluid.

Supplementary Figure 4


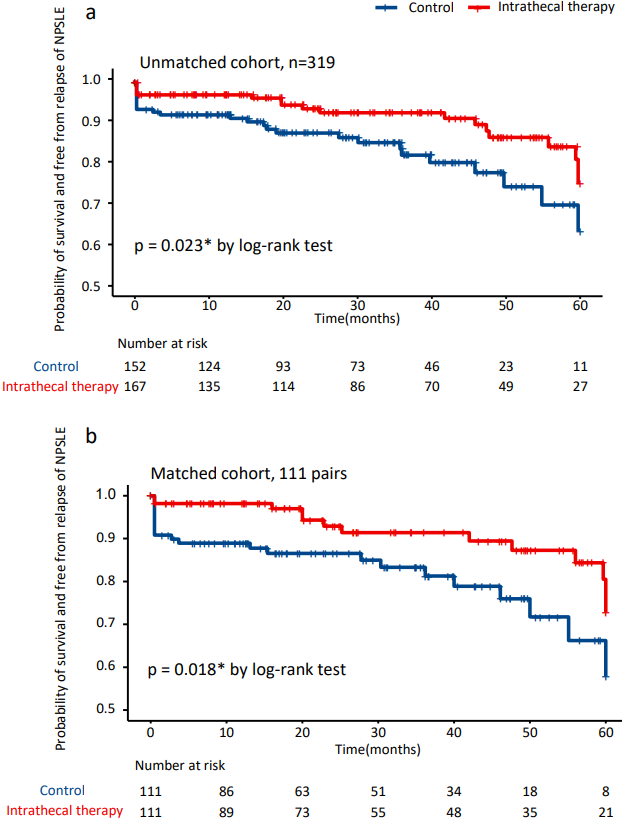


Supplementary Figure 4 The Kaplan-Meier plot of NPSLE patients during follow-up after the cases only presented headache were excluded (n=69). a. Kaplan-Meier plot of patients with NPSLE in the intrathecal treatment group or the control group in the unmatched cohort. b. Kaplan-Meier plot of patients with NPSLE in the intrathecal treatment group and the control group in the matched cohort by propensity score matching (PSM); The covariates used in PSM included sex, age, methylprednisolone pulse, SLEDAI-2K scores, psychosis. Note: NPSLE: neuropsychiatric systemic lupus erythematosus.

Supplementary Figure 5


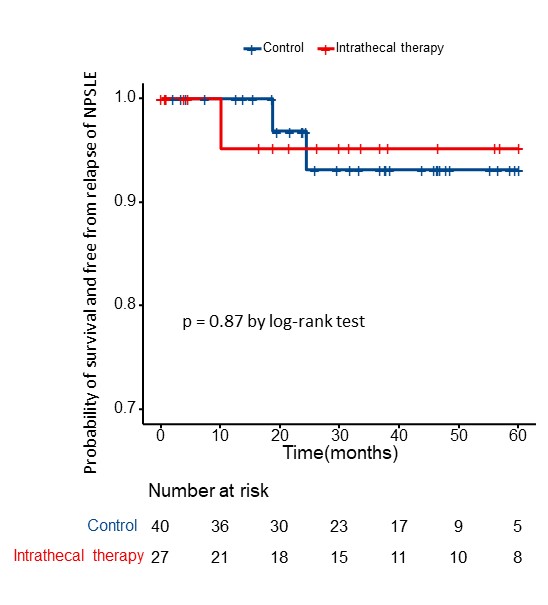


Supplementary figure 5. The Kaplan-Meier analysis plot of NPSLE patients only presented headache symptom in the two groups(n=67). Note: NPSLE: neuropsychiatric systemic lupus erythematosus.
